# Cracking the code of complex atrial tachyarrhythmias after heart transplantation: Can ultra–high-density mapping provide the key?

**DOI:** 10.1016/j.hrcr.2025.08.017

**Published:** 2025-08-21

**Authors:** Cas Teunissen, Wil Kassenberg, Marish I.F.J. Oerlemans, Mostafa M. Mokhles, Peter Loh, Astrid A. Hendriks

**Affiliations:** 1 Department of Cardiology, Heart Lung Center Utrecht, University Medical Center, Utrecht, The Netherlands; 2 Transplantation Center UMC Utrecht, Utrecht, The Netherlands; 3 Department of Cardiothoracic Surgery, Heart Lung Center Utrecht, University Medical Center, Utrecht, The Netherlands

**Keywords:** Orthotopic heart transplantation, Atrial tachycardia, Electroanatomic mapping, Radiofrequency ablation, Reentry

**Key Teaching Points**

• After orthotopic heart transplantation (OHT), ultra–high-density mapping can be valuable in accurately visualizing complex atrial tachyarrhythmias (ATs).
• Ultra–high-density mapping during sinus rhythm provides detailed insights into conduction properties between the native and transplanted atria, offering diagnostic clues for potential critical isthmuses of arrhythmias.
• Atrio-atrial connection after biatrial OHT may serve as the primary substrate for complex ATs.

## Introduction

Orthotopic heart transplantation (OHT), using either the bicaval or the biatrial anastomosis technique, is the gold standard for end-stage heart failure. Biatrial OHT involves anastomosing the donor and recipient atrial cuffs. A relatively common complication of biatrial OHT is atrial tachyarrhythmia (AT), which may include complex scar-related micro- or macroreentrant tachycardias.^1^

Ablation therapy is a possible curative option for drug-refractory AT.^2^ However, in reentrant AT, inadequate visualization and understanding of intracardiac activation sequences may hamper ablation success. Advances in electroanatomic mapping (EAM) systems and mapping catheters may enhance AT analysis and improve ablation outcomes.

We present a case of complex biatrial flutter in a patient who underwent OHT, in which ultra–high-density mapping identified the critical isthmus essential for successful ablation.

## Case report

**Figure 1:**
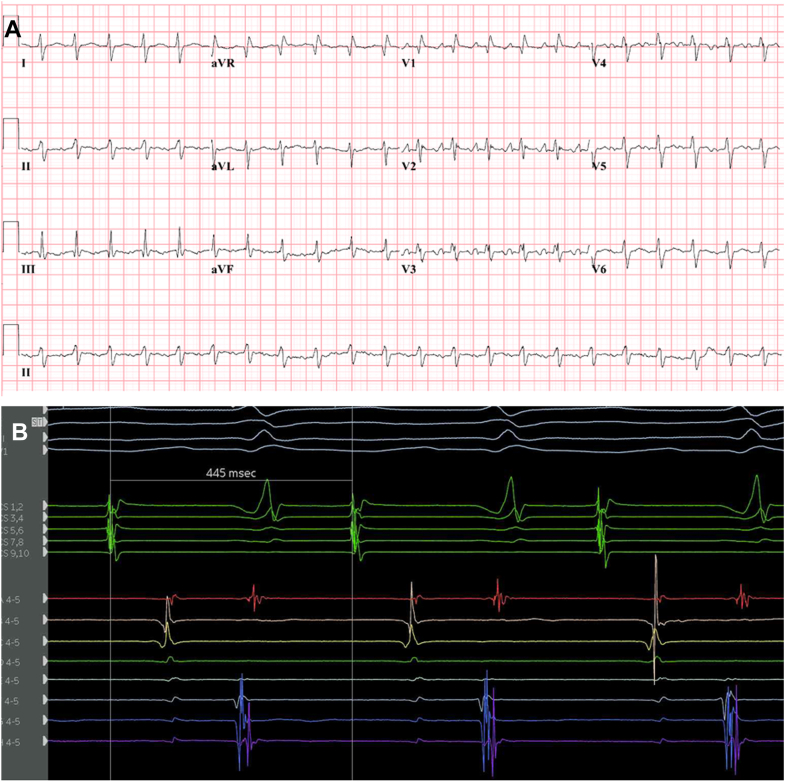
**A:** Electrocardiogram of the atrial tachyarrhythmia with a ventricular rate of 133 beats/min. **B:** Intracardial recordings of the arrythmia with a cycle length of 445 ms. CS activation was from distal (CS 1,2) to proximal (CS 9,10). The IntellaMap Orion catheter was positioned at the anastomosis of the right native and donor atria. A significant conduction delay between the donor RA and the native RA is observed. CS = coronary sinus; RA = right atrium.

A 58-year-old man with dilated cardiomyopathy underwent biatrial OHT 3.5 years ago, with surgical anastomosis as previously described.^3^ He presented with incessant AT at a ventricular rate of 133 beats/min (Figure 1), leading to tachycardia-induced impairment of left ventricular (LV) function in the transplanted heart, with an LV ejection fraction of 36%. NT-proBNP was increased to 1887 pg/mL. He was on sotalol 160 mg twice daily, but electrical cardioversion was unsuccessful under this regimen.

An electrophysiology study was performed under deep sedation. EAM was conducted using an ultra–high-density mapping system (Rhythmia, Boston Scientific). A multielectrode catheter (Inquiry, Abbott) was positioned in the coronary sinus (CS). Mapping was performed using a 64-electrode minibasket catheter (IntellaMap Orion, Boston Scientific). Radiofrequency ablation was performed with a 4-mm-tip open-irrigated ablation catheter, incorporating contact force and local impedance sensing (IntellaNav StablePoint, Boston Scientific).

**Figure 2:**
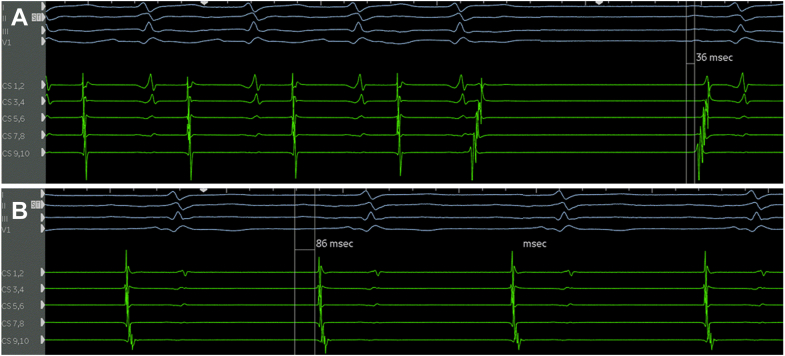
Atrial activation patterns during SR before and after ablation of the right-sided AAC. **A:** Before ablation. Transition of the atrial tachycardia to SR after a premature atrial complex. During SR, CS activation was from proximal (CS 9,10) to distal (CS 1,2). **B:** After ablation, a change in CS activation sequence and a prolonged interval from the onset of the P wave to CS activation were observed compared with preablation (86 ms vs 36 ms). After ablation, sinus node activation from the native heart reached the AV node from the donor heart via conduction through the remaining AAC at the LA anastomosis, suggesting conduction block over the right-sided AAC. AAC = atrio-atrial connection; AV = atrioventricular; CS = coronary sinus; LA = left atrium; SR = sinus rhythm.

**Figure 3:**
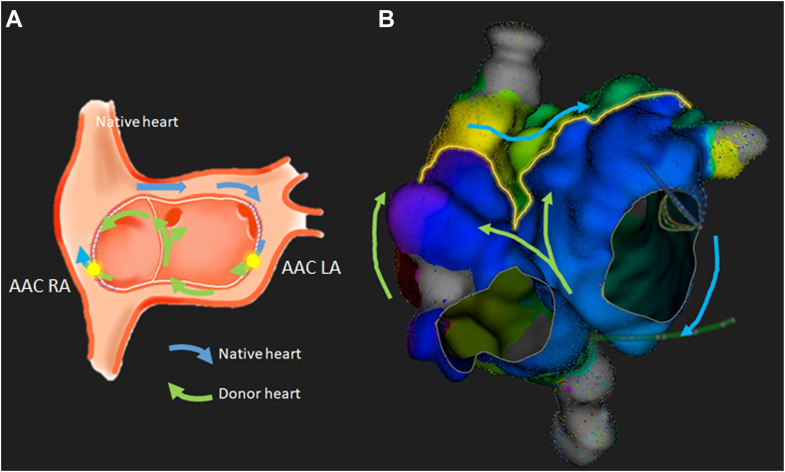
Schematic representation (**A**) and Rhythmia map (**B**) of activation sequence of biatrial flutter. Atrial activation in the donor RA propagates through the first AAC located laterally, subsequently reaching the posterior region of the native RA. The wavefront then traverses the Bachmann bundle to enter the native LA. Within the lateral region of the native LA, conduction proceeds via a second AAC connecting the native LA and donor LA, resulting in activation of the CS in a distal-to-proximal direction. Activation of the interatrial septum occurs from inferior to superior, involving both the donor LA and the donor RA. The activation wave then ascends along the superior aspect of the donor RA and reenters the circuit via the initial lateral AAC, thereby completing a macroreentrant circuit. This constitutes a biatrial flutter circuit involving all 4 atrial “chambers”: the donor and native RAs and LAs. AAC = atrio-atrial connection; CS = coronary sinus; LA = left atrium; RA = right atrium.

**Figure 4:**
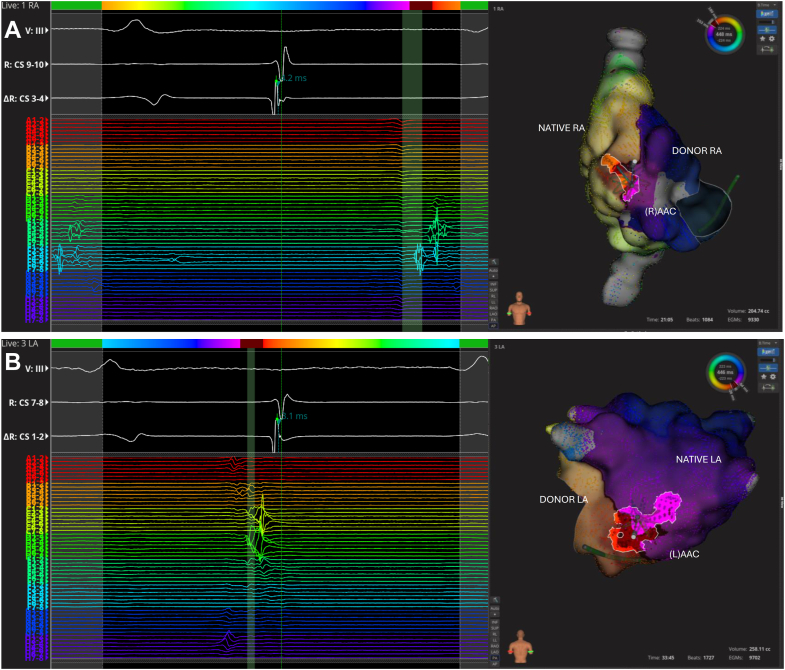
Intracardiac signals recorded with the IntellaMap Orion catheter at the right-sided AAC (**A**) and left AAC (**B**). Particularly at the right-sided AAC, low-voltage fractionated electrograms were observed. Ablation at this site resulted in termination of the tachycardia. AAC = atrio-atrial connection; LA = left atrium; RA = right atrium.

At the beginning of the electrophysiology study, the patient was in AT with a cycle length of 445 ms, interspersed with brief episodes of sinus rhythm (SR). During AT, CS activation was distal to proximal (Figure 1), whereas during SR, it was proximal to distal (Figure 2A). After a transseptal puncture, EAM was performed in the left atrium (LA) and right atrium (RA). Mapping revealed complex biatrial flutter with a figure-of-eight activation pattern (illustrated in Figure 3 and Supplemental Video 1), using conduction gaps at the anastomoses between the native and donor atria at both the lateral RA and the lateral LA (Figure 4). Radiofrequency ablation at the corridor of the RA anastomosis successfully terminated the biatrial tachycardia, rendering it noninducible. After ablation, during SR, a change in CS activation sequence and a prolonged interval from the onset of the P wave to CS activation were observed compared with preablation (Figure 2B). The earliest activation during SR was recorded high in the native RA at the site of the native sinus node. After ablation of the conduction gap at the RA anastomosis, sinus node activation from the native heart reached the atrioventricular node from the donor heart via conduction through the remaining gap at the LA anastomosis, suggesting conduction block over the RA anastomosis.

After 1 year of follow-up, there was no recurrence of AT, and LV function showed subsequent improvement, with an ejection fraction of 50%. Six months after ablation, NT-proBNP decreased to 332 pg/mL and normalized 3 months later to 96 pg/mL.

## Discussion

We present a case of complex AT after OHT, effectively analyzed and visualized using ultra–high-density EAM. The incidence of AT after heart transplantation is reported to be 7%–9%, with most cases occurring in the early postoperative period.^4^ Late-onset AT in patients who underwent OHT is primarily owing to cavotricuspid isthmus–dependent atrial flutter or scar-mediated reentry.^4^^,^^5^ Pharmacological treatment is often limited by intolerance to antiarrhythmic drugs.

Conduction gaps between the native and donor atria, also referred to as atrio-atrial connections (AACs), have been reported in up to 21% of patients who underwent OHT.^6^ The formation of AACs may be influenced by a patient’s immune advantage, characterized by the absence of donor-specific antibodies.^5^ AACs can serve as isthmuses for reentrant circuits because of relatively slow conduction in these regions. Consequently, AACs may serve as potential ablation targets. However, ablation of AACs may result in disconnection between the native and donor atria, potentially leading to chronotropic incompetence. In our case, ablation of the left AAC may have been a more optimal strategy, as it could have preserved a more physiological atrioventricular conduction during SR. Our standard procedural workflow involves creating an activation map during SR to assess atrial conduction. However, this was not feasible in the present case because of the incessant nature of the AT. Given its relatively straightforward accessibility, we proceeded with ablation of the right-sided AAC. This procedural choice—and its subsequent clinical implications—represents a learning point of this case.

Ultra–high-density EAM provides exceptionally high spatial and temporal resolution by collecting tens of thousands data points per map.^7^ The minielectrode configuration of the Intellamap Orion mapping catheter and the signal processing algorithms of the mapping system excel at identifying low-amplitude, fractionated, and multicomponent electrograms. It improves near-field signal resolution and effectively reduces far-field noise.^7^ The resulting fine spatial granularity allows for improved differentiation between active conduction, passive propagation, and areas of conduction block.^8^ In our case, these features were instrumental in delineating the arrhythmogenic substrate and guiding targeted ablation.

## Conclusion

After OHT, ultra–high-density EAM proved instrumental in uncovering complex tachycardia waveforms, thereby facilitating successful ablation. Additionally, it provides valuable insights into atrial conduction during SR.

## Disclosures

The authors have no conflicts of interest to disclose.
