# Supplementary material for: Cracking the code of complex atrial tachyarrhythmias after heart transplantation: Can ultra–high-density mapping provide the key?
Source: HeartRhythm Case Rep. 2025 Aug 21;11(11):1166–70. doi: 10.1016/j.hrcr.2025.08.017 (PMC12666923; doi:10.1016/j.hrcr.2025.08.017)
Supplement: Supplementary Material [file mmc2.docx]

Movie 1 Legend

Activation sequence of the biatrial flutter.

Atrial activation in the donor RA propagates through the first AAC located laterally, subsequently reaching the posterior region of the native RA. The wavefront then traverses Bachmann’s bundle to enter the native LA. Within the lateral region of the native LA, conduction proceeds via a second AAC connecting the native and donor LA, resulting in activation of the CS in a distal-to-proximal direction. Activation of the interatrial septum occurs from inferior to superior, involving both the donor LA and donor RA. The activation wave then ascends along the superior aspect of the donor RA and re-enters the circuit via the initial lateral AAC, thereby completing a macro reentrant circuit. This constitutes a biatrial flutter circuit involving all four atrial “chambers”: the donor and native RA and LA.

AAC: atrio-atrial connection, CS: coronary sinus, LA: left atrium, RA: right atrium
